# A compact and miniaturized implantable antenna for ISM band in wireless cardiac pacemaker system

**DOI:** 10.1038/s41598-021-04404-3

**Published:** 2022-01-07

**Authors:** Yang Feng, Zhaonan Li, Lin Qi, Wanting Shen, Gaosheng Li

**Affiliations:** College of Electrical and Information Engineering, Hunan University, Changsha, 410082 China

**Keywords:** Electrical and electronic engineering, Biomedical engineering

## Abstract

A tiny and compact implantable antenna for wireless cardiac pacemaker systems is designed. The antenna works in the Industrial Scientific Medical (ISM) frequency band (2.4–2.48 GHz). The size of the antenna is greatly reduced with the adoption of a high dielectric constant medium and a folded meander structure. The volume of the antenna is 4.5 mm^3^, and the size is only 3 mm × 3 mm × 0.5 mm. Based on the literature research, it was found that the design was the smallest among the same type of implanted antenna. The antenna is optimized and loaded with a defective slotted structure, which improves the efficiency of the overall performance of the antenna and thus the gain thereof. The antenna maintains good impedance matching in the ISM frequency band, covering the entire ISM frequency band. The actual bandwidth of the antenna is 22%, with the peak gain of − 24.9 dBi. The antenna is processed and manufactured in such a manner that the simulation keeps consistent with the actual measurement. In addition, the specific absorption rate of the antenna is also evaluated and analyzed. The result shows that this kind of antenna is the best choice to realize the wireless biological telemetry communication in the extremely compact space of the wireless cardiac pacemaker system.

## Introduction

The rapid development of electronic technology has promoted the birth of a new generation of pacemakers and embedded cardioverter defibrillators. As a communication carrier for wireless electronic medical equipment, antennas are playing an increasingly important role. At present, nearly 3 million people in the world are using pacemakers^1^. The pacemaker system realizes the function of wireless communication, which could improve the comfort of the patient and power supply safety of the device.

**Figure 1:**
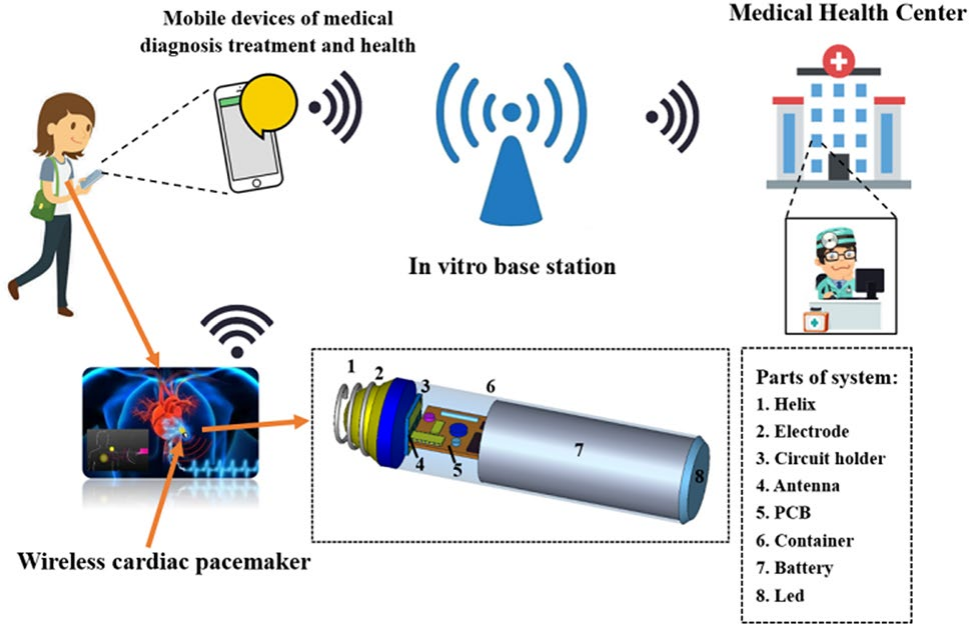
Schematic diagram of application scenarios of wireless cardiac pacemaker system (similar to Nanostim).

As shown in Fig. 1, the wireless cardiac pacemaker system provides the patients with good integrated and convenient medical services through antennas. When the wireless pacemaker system satisfies the normal operation of the cardiac pacemaker, real-time monitoring and protection become more important. Such being the case, the miniaturization, working life and medical communication level of the pacemaker system matter most^2^. Therefore, the design and research of implantable antennas used in wireless cardiac pacemaker systems is still an important research topic.

With the dedicated frequency resources for medical electronics and radio information transmission allocated bythe International Telecommunication Union (ITU), the radio transmission of medical implants is granted with a reliable spectrum resource. There are mainly three frequently used operating frequency bands for implantable antennas, namely the medical implantable communication service frequency band (MICS, 402–405 MHz), the wireless medical telemetry service frequency band (WMTS, 1.395–1.4 GHz), and the industrial, scientific, and medical frequency bands (ISM, 433–434 MHz, 902–908 MHz, 2.4–2.48 GHz, 5.715–5.875 GHz). In addition, some foreign regions have also authorized ultra-wideband frequency bands for high-quality transmission (UWB, 3.1–10.6 GHz).

Research teams around the world have conducted researches on miniaturized implantable antennas. The implantable antennas issued in public prints are developing towards diversification, functionalization, convenience and miniaturization. The most important thing about the antenna used in the pacemaker system is how to make it smaller in size and volume. The smaller the device is, the better it can alleviate the rejection of implants and relieve the pain of patients^3^. designed an implantable antenna that serves the MICS frequency band for cardiac pacemakers. The antenna adopts a conformal meander structure with a radius of 4.73 mm and a length of 20.5 mm. The antenna works in the MICS frequency band with a gain of − 32 dBi. In^4^, an implantable conformal antenna loaded with an open loop resonator (CSRR) was designed for use in a leadless pacing system. The design has a compact design and works at 2.45 GHz. The antenna has a peak gain of − 35 dBi, but it is too large in size. Literature^5^ designed a miniaturized broadband implantable antenna, which realized the miniaturization of the antenna by opening several different slots on the radiation patch and introducing short-circuit pins. In^6^, a coplanar waveguide fed (CPW) patch implanted antenna with a size of 24 × 22 × 0.07 mm^3^ was designed. The antenna gain designed in this research was − 19.7 dBi, with a bandwidth of 24%. Although it had a wider bandwidth and greater gain, it was at the expense of the size^7^. introduced a broadband implantable antenna suitable for implantable medical devices. The antenna adopteda symmetrical geometric structure as the radiation patch, which has good radiation performance. In^8^, the author realized the miniaturization of the antenna by using the meandering radiation patch and loaded short-circuit pin, and slotted the antenna ground plane. The antenna was small in size and worked in multiple frequency bands. Recently, an ultra-small implantable antenna was proposed in^9^. The antenna was used in the pacemaker system to serve the ISM frequency band with the size of 3 × 4 × 0.5 mm^3^. The simplified structural design of the antenna was easy for system integration. However, the peak gain of the antenna was only − 25.95 dBi.

This paper introduces an ultra-compact implantable antenna, which serves biomedical telemetry and is applicable to wireless cardiac pacemaker systems. The volume of the proposed antenna is only 4.5 mm^3^, and 3 mm × 3 mm × 0.5 mm in size. Through literature review, it is found that the antenna designed in this paper is the smallest among the implanted antennas of the same type and structure, which is consistent with the popularity of miniaturization of implanted electronic devices. In this paper, through optimization analysis of the antenna's reflection coefficient, current distribution, radiation efficiency, specific absorption rate (SAR) and other performances, it is verified that the designed antenna meets the requirements of radiation performance, design and effect while being very small in size. The paper introduces the methodology in “Methodology” section, describes the performance analysis and discussion of the implantable antenna in “Analysis and discussion” section, and makes a summary in the last section.

## Methodology

### Antenna structure design

The planar antenna with a zigzag structure has the advantages of compact structure, small size, light weight, and easy processing. Combined with the spatial structure of the implantable electronic device, the implanted antenna with a flat zigzag structure is a good choice.

In the antenna design process, the length of the bus will affect its resonance characteristics. The increase in length will extend the path of the current, so that the resonance frequency will shift to low frequencies. The antenna bus calculation formula is shown in (1):

1 $$L \approx \frac{c}{{4f\sqrt {\varepsilon_{r} } }},$$

In formula (1), L is the bus length of the antenna, f is the resonant frequency of the antenna, and c is the speed of electromagnetic waves in vacuum.

The working environment of the implantable antenna is in a mixed medium, not a free space. After implantation in biological tissue, the effective dielectric constant will change to a certain extent. According to the Lichtenecker formula (formula (2)), the effective dielectric constant is related to the dielectric constant and volume fraction of various mixed media. After analyzing the parameters, we replace $\varepsilon_{r}$ in formula (1) with $\varepsilon_{{{\text{eff}}}}$. The dielectric constant obtained in this way will be more accurate.

2 $$\ln (\varepsilon_{{{\text{eff}}}} ) = v_{2} \ln (\varepsilon_{1} ) + v_{1} \ln (\varepsilon_{2} ),$$

In the formula (2), $v_{1}$, $v_{2}$ are the relative volume fractions of the two substances; $\varepsilon_{1}$, $\varepsilon_{2}$ are the relative permittivity of the two substances.

**Figure 2:**
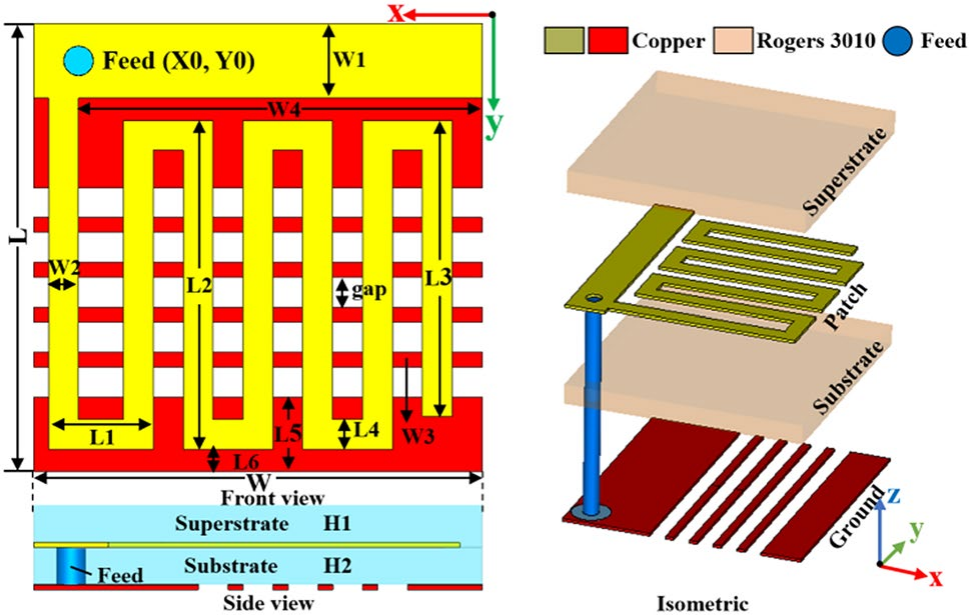
Implantable antenna structure diagram (Unit: mm).

In the process of designing a wireless cardiac pacemaker system, antennas with the simple meander structure are usually selected for the convenience of manufacturing. The complex structure of the antenna form will cause greater trouble in processing^9^. The overall structure of the antenna designed in this paper is simple. Figure 2 presents the planar structure diagram and the three-dimensional structure expansion diagram of the antenna, respectively.

To meet the needs of miniaturized antenna structure and the rational use of the limited space of the pacemaker system, the antenna is designed to be 3 × 3 × 0.5 mm^3^ in size. The size of this design is the smallest among similar antennas in accordance with the existing literature. The radiating layer of the antenna adopts the patch with a zigzag structure to expand the current path, so as to achieve miniaturization. The short branches of the radiating patch play the role of guiding current and optimizing impedance matching. In addition, the antenna employs a high dielectric constant substrate to further reduce its size. The ground plane is loaded with a slotted structure, which is equivalent to the effect of capacitive loading. The ground plane is slotted to enhance the electric field strength of the antenna, thereby improving its radiation efficiency thereof. In order to protect the radiation safety of the human body and prevent the high dielectric properties of the biological tissue structure from damaging the performance of the antenna, this design uses the dielectric plate which is consistent with the base as the covering layer of the radiation patch, so as to have a good protective effect^10^. The dielectric constant (εr) of the antenna substrate is 10.2, and the loss tangent (tan δ) is 0.0023. We selected the Rogers 3010 owing to its high permittivity. This paper mainly focuses on the angle of antenna design. Antennas are usually embedded in implantable electronic medical equipment during application. More biocompatibility issues should be considered at the level of electronic systems. Usually, the electronic equipment used in the antenna will add a layer of biocompatible material film. Such considerations can meet the needs of biocompatibility. In addition, in order to prevent the occurrence of biocompatibility incidents in the antenna design, some treatments are also done to prevent harm to the human body. In order to meet the requirements of biocompatibility, the processed antenna is coated with a ceramic aluminum oxide film on the surface of its structure. The thickness of the antenna substrate is reduced as much as possible to 0.25 mm. The antenna is coaxial-fed with the position of the feeding point is x = 2.7 mm and y = 0.25 mm. The specific parameter size of the antenna structure is shown in Table 1.

**Table 1:** The parameters of the proposed antenna (Unit: mm).

Variable	Value	Variable	Value	Variable	Value
L	3	L5	0.5	W3	0.1
L1	0.7	L6	0.15	W4	2.7
L2	2.2	W	3	H1	0.25
L3	1.98	W1	0.5	H2	0.25
L4	0.2	W2	0.2	gap	0.2

### Simulation environment and test environment settings

**Figure 3:**
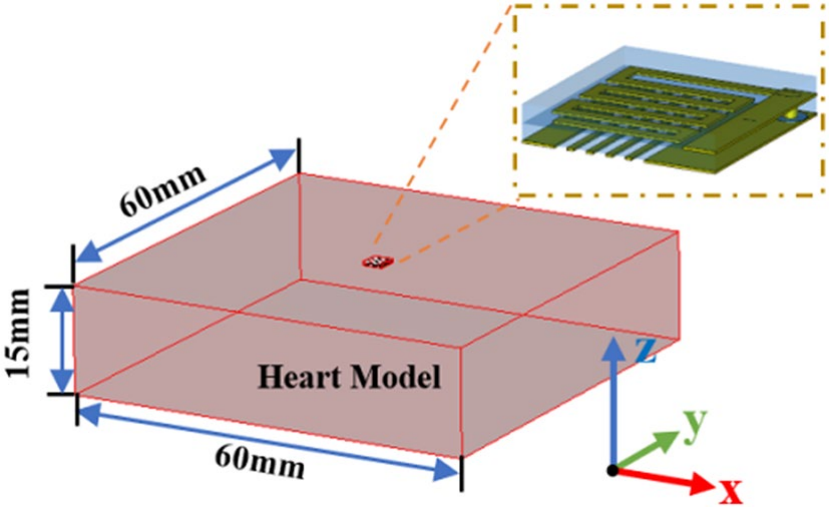
Schematic diagram of antenna implantation simulation heart tissue model.

Human tissue has characteristics of dispersion, and the dielectric change of the human body has little effect on the performance of the implanted antenna^11^. The implanted antenna is modeled and optimized in the equivalent model of the typical human heart. The conductivity of the heart tissue at 2.45 GHz is 2.215 and the dielectric constant is 54.918. Antenna implantation depth is 3 mm. The schematic diagram of the antenna implanted heart tissue model and its structure information are shown in Fig. 3.

**Figure 4:**
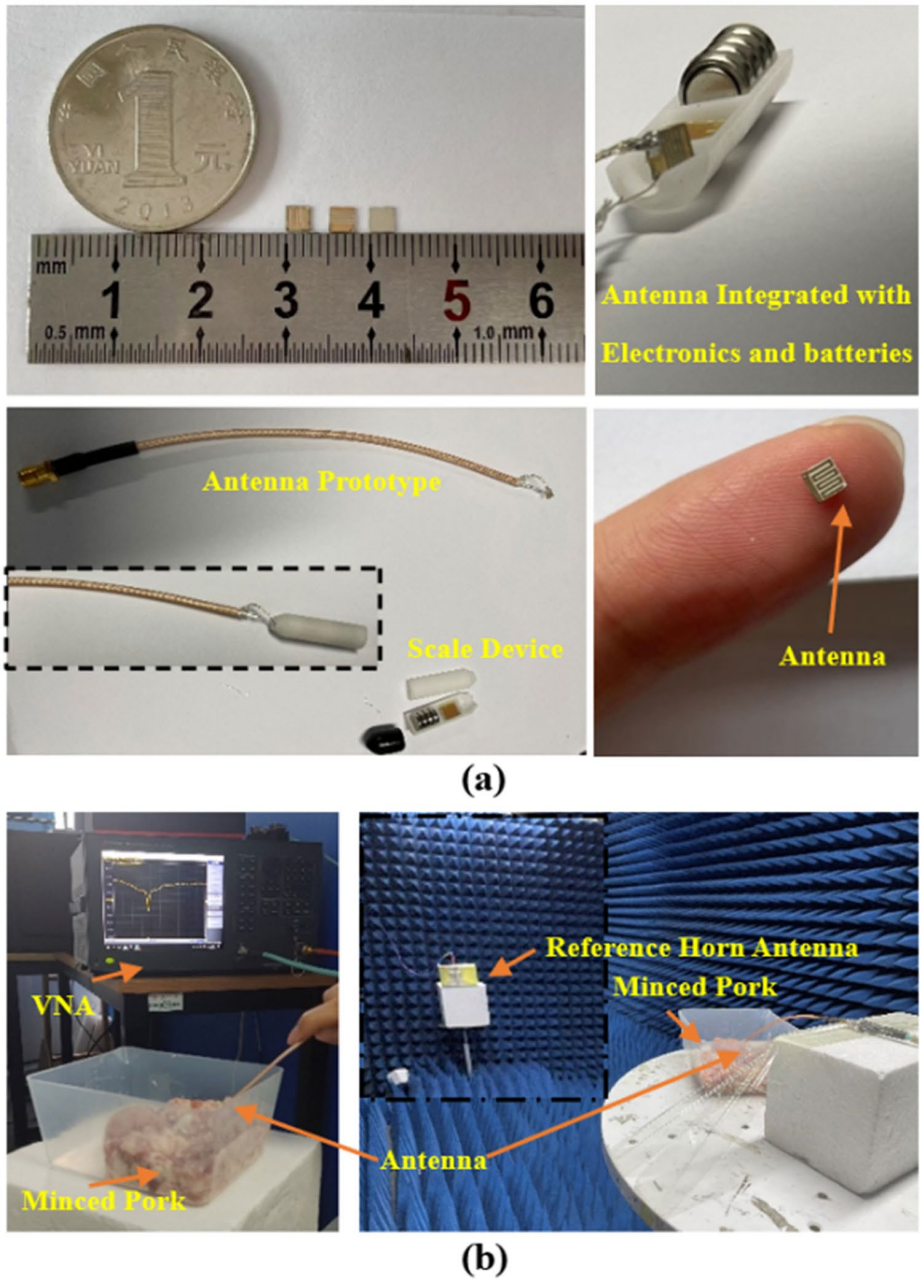
Antenna and test environment. (**a**) Antenna components and 3D printed cardiac pacemaker system models. (**b**) Antenna test S parameters and gain environment layout.

The actual test scenario and the processed antenna and its system are shown in Fig. 4. The antenna is manufactured and processed by etching technology. The fabricated antenna, circuit, and battery are integrated into a 3D printed imitation pacemaker system device. This equipment is used to test the working condition of the antenna in the system equipment. Minced pork was used as the antenna implantation environment. According to the literature, the dielectric properties of ground pork are similar to those of the human body^12^. The antenna S parameter test adopts the E5063A vector network analyzer (VNA). The radiation characteristics of the antenna were tested in the minced pork. The designed antenna is compared with a standard horn antenna to obtain the best gain.

## Analysis and discussion

**Figure 5:**
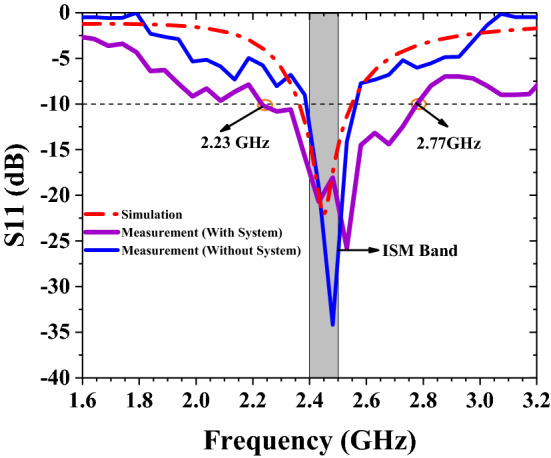
The simulated and measured return loss of the antenna with or without a pacemaker system.

S11 of the antenna is obtained in this paper by adjusting the coaxial-fed position and optimizing the structure of the implanted antenna. This paper refers to the physical design and simulates the structure of a pacemaker system to test the interference of the system to the antenna. The 3D-printed imitation pacemaker system tries its best to recreate the actual working environment so as to better test the performance of the antenna and make the test results more accurate. The antenna simulation and actual test results with or without pacemaker system equipment are shown in Fig. 5.

According to the comparison in Fig. 5, the simulation results are basically consistent with the actual test results. The antenna is more stable and anti-interference. The working bandwidth of the antenna is sufficient to cover the ISM band. The return loss at 2.45 GHz is less than − 10 dB, which shows that almost all of the antenna radiated power is transmitted to the medium. When the antenna was separately put into the minced pork for testing, the impedance matching was satisfying which was basically consistent with the simulation results. When the antenna was integrated into an analog system, there were some changes in the performance, and the bandwidth had been broadened to a certain extent. This is caused by manufacturing errors as well as partial coupling between the device and the antenna. The impedance bandwidth of the antenna tested in the pacemaker system is 2230–2770 MHz, and its relative bandwidth is 22%. The wider bandwidth is conducive to the antenna work in the complex and changeable heart tissue. As the human body may change in physiological performance, leading the antenna to have a detuning effect. The application of wide bandwidth is beneficial to overcome the drift of the working frequency caused by the antenna detuning effect.

**Figure 6:**
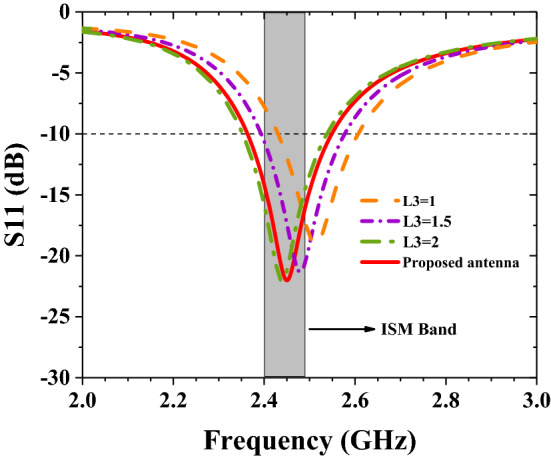
The effect of antenna L3 length on S11.

The bus length of the antenna determines the resonant frequency of the antenna. Figure 6 shows the influence of different lengths of the reduced meandering curve L3 on the resonant frequency of the antenna. It can be seen from the simulation results that the longer the length of L3, the more the resonance frequency shifts toward the low frequency direction. This result is consistent with the result of theoretical analysis, that is, the length of the entire radiating patch is inversely proportional to the resonance frequency. According to the optimization analysis, when L3 = 1.98 mm, the resonance frequency point falls to 2.45 GHz, which achieves the desired effect.

**Figure 7:**
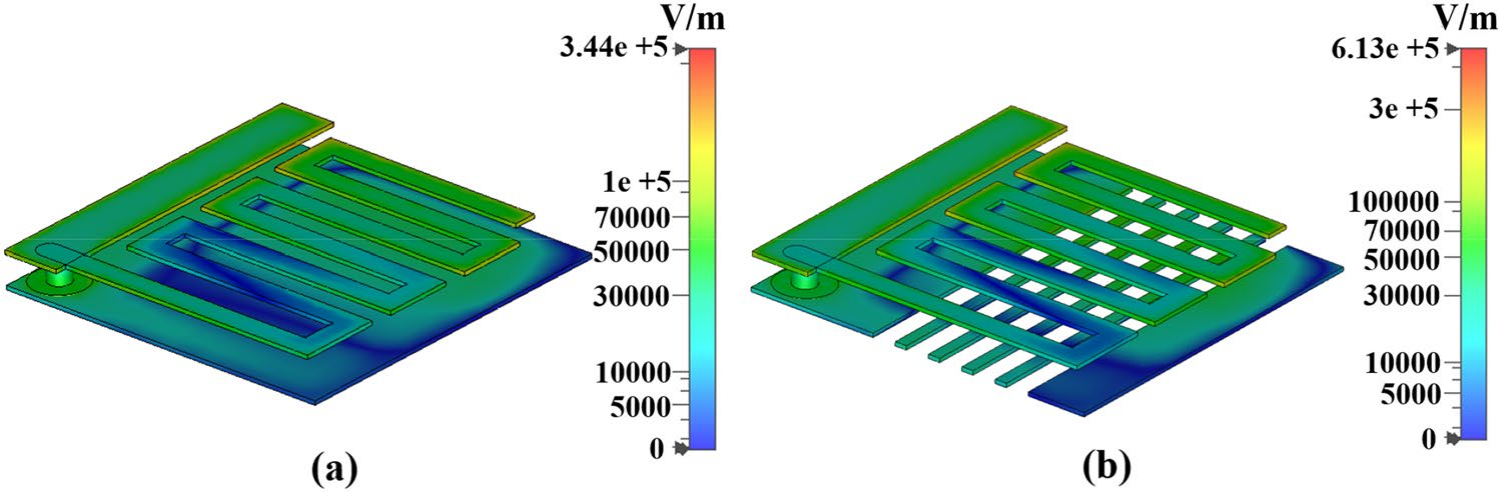
The influence of floor slotting on antenna electric field distribution (**a**) intact ground (**b**) slotted ground.

The electric field distribution of the slotted and unslotted antenna grounding plane is shown in Fig. 7. The slotted ground contributed partly to the radiation performance of the entire antenna structure. On the one hand, slotting at the ground plane changes its current distribution of the ground plane. On the other hand, it amplifies the capacitive coupling effect, thus increases the electric field strength, and further improves the gain of the antenna.

The antenna obtains an optimal radiation effect by optimizing the slot width, spacing and number of slots. Figure 7a shows that the maximum electric field strength of the antenna is 3.44 × 10^5^ V/m without treatment. Figure 7b shows that the maximum electric field strength of the antenna is 6.13 × 10^5^ V/m after optimizing the slotting of the ground plane. The designed antenna has a low operating frequency and works in an environment with large dielectric loss. The gap magnitude of the slot is small relative to the frequency band, and the gap will not increase its backward radiation. On the contrary, it can be seen from the electric field strength that the space field superimposition effect of the two-layer structure is significant. The designed ground slot can form a leaky wave radiator, which helps to improve the overall performance of the antenna. It can be verified that the slotted structure of the ground plane improves the radiation performance of the antenna and increases the electric field strength of the antenna.

**Figure 8:**
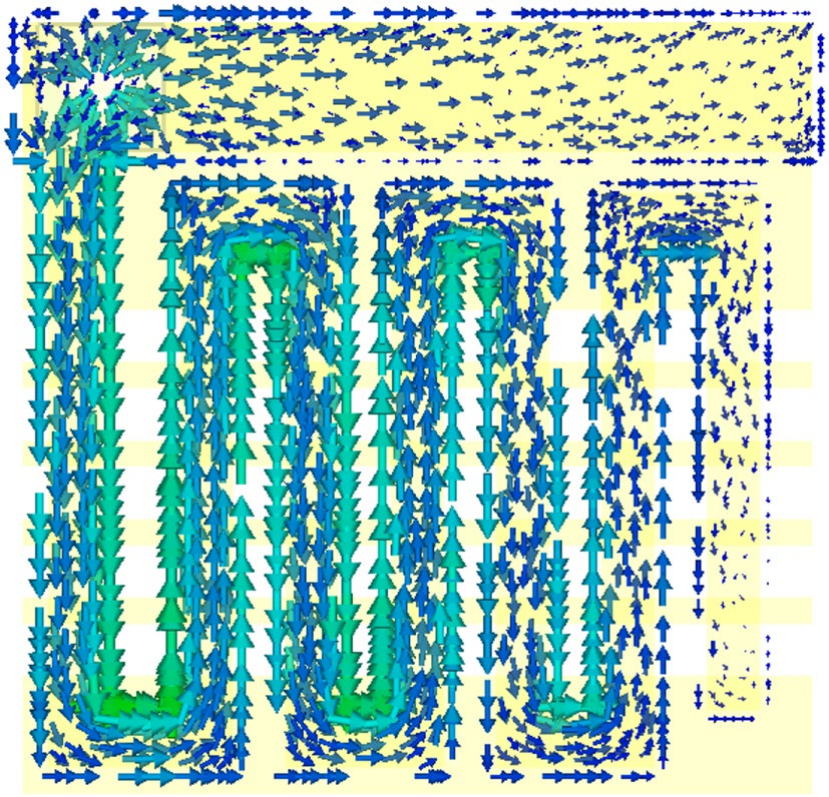
Antenna current distribution diagram (f = 2.45 GHz).

Figure 8 shows the current distribution of the target antenna radiation patch at the resonance frequency. The figure clearly shows that the current direction of the antenna flows from the feed point in the same direction. Therefore, the antenna resonates in a quarter-wavelength monopole mode.

**Figure 9:**
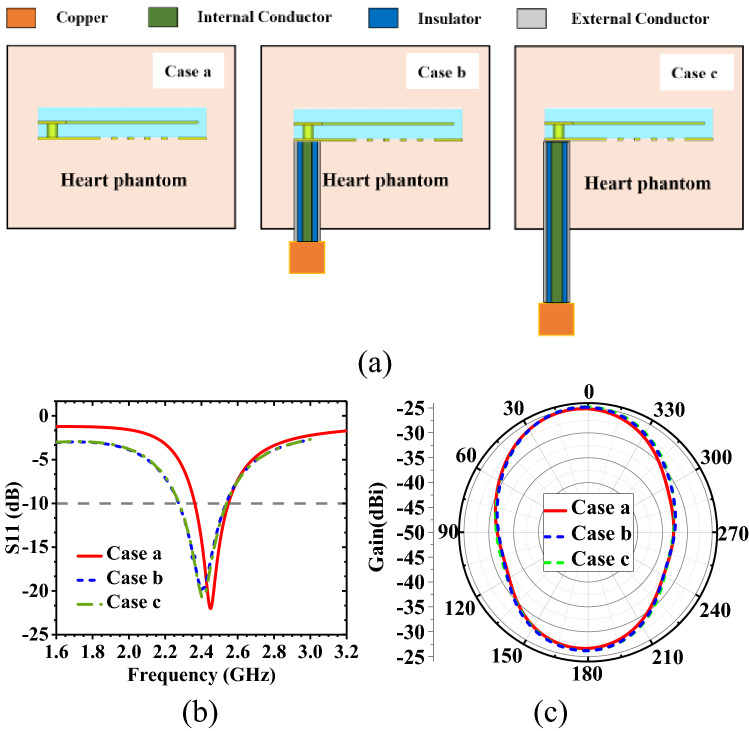
Coaxial cable effects on the antenna performance (f = 2.45 GHz). (**a**) Model in different cases. (**b**) S11 of antennas in different cases. (**c**) Radiation patterns of antennas in different cases.

The antenna measurement is carried out by using radio frequency cables. Considering that the coaxial cable may interfere with the performance of the antenna, we set three scenarios in the simulation model (The scenarios are shown in Fig. 9a): Case a: the antenna model without cables, Case b: the antenna model with short cable and Case c: the antenna model with long cable. Observing the reflection coefficient in Fig. 9b, it can be seen that S11 is slightly deteriorated, which is due to the influence of the coupling effect. In the same situation, as shown in Fig. 9c, the peak gain of the antenna has a deviation of about 0.2 dBi. It can be seen that the coaxial cable has a relatively small impact on the performance of the implantable antenna. The impedance characteristics and radiation characteristics of the antenna remain basically unchanged, which can indicate that the coaxial cable has little effect on the impact. The results also show that the designed antenna has better robustness.

**Figure 10:**
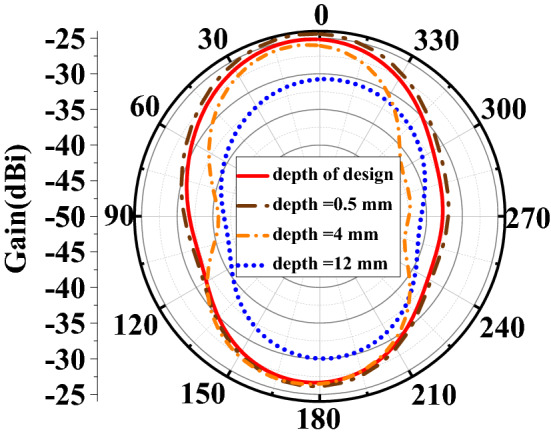
the effects of implantation depth on the antenna performance (radiation pattern).

The implantation depth of the implanted antenna has a certain influence on the performance of the antenna. Usually, the deeper the implantation, the greater the loss and the lower the gain of the antenna. As shown in Fig. 10, we have also verified this statement.

**Figure 11:**
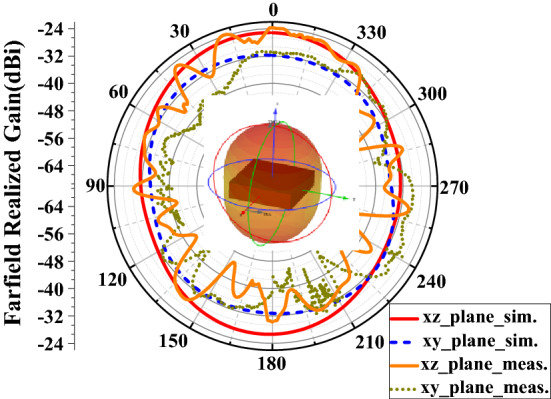
Gain of the proposed capsule antenna (f = 2.45 GHz).

In the entire field of implantable antenna design, the final working gain of the antenna is generally low. Due to the limitation of the size of the antenna itself, the radiation efficiency of the antenna is greatly reduced^13^. As shown in Fig. 11, the antenna’s peak gain is − 25 dBi. It can be seen in the figure that the implantable antenna has been achieved with such characteristics as omnidirectional radiation. Since the heart tissue environment of the wireless cardiac pacemaker system is complex and changeable, such characteristics as omnidirectional radiation can realize information transmission in all directions.

**Figure 12:**
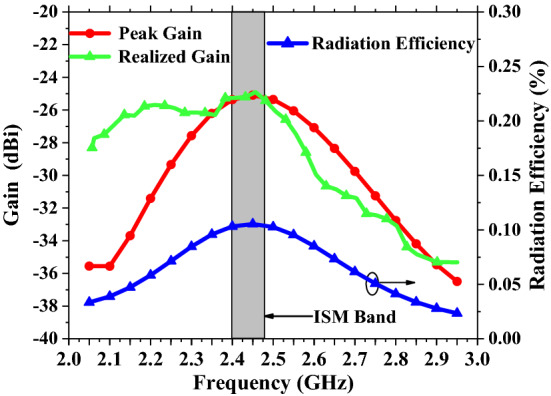
Graph of implantable antenna gain and radiation efficiency vs. frequency.

Figure 12 shows the radiation efficiency and peak gain of the proposed antenna in the 2–3 GHz frequency band. In the ISM frequency band, the radiation efficiency of the target antenna is approximately 0.1%. There is a coupling effect between the antenna and human tissue, and the radiation efficiency is greatly reduced. Generally, the radiation efficiency of implanted antennas is less than 1%^14^. Comparing the peak gain with the realized gain, it can be seen that the desired gain is obtained in the ISM frequency band, with the realized gain of − 24.9 dBi. The gain is measured in minced pork with the similar environment. Though there is a certain difference with the simulated heart tissue model, the structure has no significant difference. In addition, there are some test errors in the actual measurement environment and fabrication, but the overall situations are basically consistent.

The antenna generates a certain amount of radiation in the process of wireless information transmission. In order to ensure the safety of human tissues and organs, the radiation power of the antenna needs to be within a limited and controllable range. Only in this way can the electronic equipment serve the human body while ensuring human safety and health. The IEEE C95.1-1999 standard stipulates that the peak average specific absorption rate (SAR) of the human body cannot exceed 1.6 W/kg and 2 W/kg in 1 g tissue and 10 g tissue respectively^15^. Supposing that the input power is 1 W, the maximum average SAR value of 1 g tissue is 32.3 W/kg, and the maximum average SAR value of 10 g tissue is 10.8 W/kg, which is far beyond the standard specified by IEEE. In order to keep the SAR within a safe and controllable range, the maximum allowable input power of the antenna should not exceed 62.5 mW. But for implantable antennas, 25 µW is the limit value of input power, which is much lower than the value calculated in this design^16^. The simulation analysis is intended to make the SAR of the implanted antenna meet the standard. Within the scope of human radiation safety control, the maximum allowable power of 1 g and 10 g tissue is 62.5 mW and 147.7 mW respectively. According to the above calculation, it can be concluded that the SAR value complies with IEEE regulations. Table 2 shows the detailed SAR value and maximum allowable input power of the antenna designed in this paper.

**Table 2:** Peak spatial average SAR (input power = 1 W) and maximum allowable input power.

Frequency (GHz)	MAX SAR(W/kg)		1 g allowable input power (mW)	10 g allowable input power (mW)
	1 g—avg	10 g—avg		
2.45	32.3	10.8	62.5	147.7

**Figure 13:**
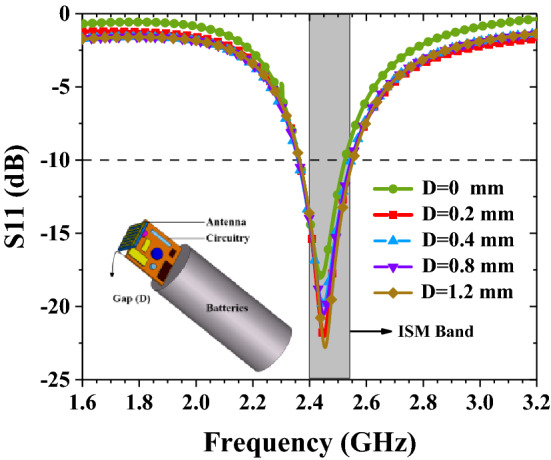
Coupling effects due to metallic components of the system on the implantable antenna performance.

Due to the presence of the device circuitry close to the antenna, there is a coupling between the device and the antenna. The battery and electronic components consider the use of perfect electrical conductor (PEC) materials, and the circuit PCB is manufactured on Roger 3010. We build a model, set the gap between the antenna and the electronic device as D, analyze and determine the best gap. Finally, we get an optimum gap through simulation analysis. As shown in Fig. 13, in order to avoid degradation of antenna performance, the best gap between the circuit device and the antenna is 0.2 mm.

Due to the loss of human tissue, the gain of the antenna is very low, which results in the short communication distance of the implanted antenna. In order to determine the communication performance of the designed antenna, its communication link needs to be analyzed. In calculating the link power budget, the designed implanted antenna is used as the transmitter (Tx), and the linearly polarized monopole antenna in free space is used as the receiver (Rx). In the communication process, the impedance matching of the transmitting and receiving antenna is good, ignoring the loss caused by the antenna mismatch. Table 3 lists the detailed parameters related to the calculation of the link budget analysis of the designed antenna.

**Table 3:** Communication link calculation related parameters.

**Transmitter**	
Frequency (GHz)	2.45
Tx power (dBm)	− 40
Tx antenna Gain (dBi)	− 24.9
**Receiver**	
Rx antenna Gain (dBi)	2.15
Ambient temperature (K)	293
Boltzmann constant	− 1.38 × 10^–23^
Noise power density	− 199.95
**Signal quality**	
Bit rate (Mb/s)	7.0
Bit error rate	1.0 × 10^–5^
E_b_/N_0_ (ideal-BPSK) (dB)	9.6
Coding gain (dB)	0
Fixing deterioration (dB)	2.5

**Figure 14:**
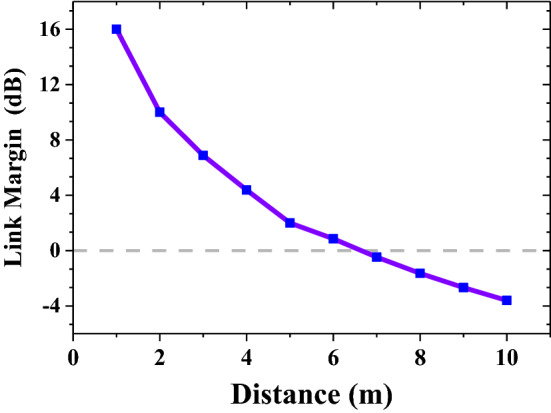
The communication link budget of the antenna at 2.45 GHz.

The communication link margin (LM) can be calculated using the relationship given in^17^. The detailed link margin is shown in Fig. 14. In order for the communication link to meet the requirements, the link margin must be greater than 0. When the input power meets the SAR safety standard, the designed implantable communication can achieve reliable communication within 6 m.

The antenna designed in this paper satisfies the needs of miniaturization and performance improvement. Table 4 shows the parameter and performance comparison between the target antenna and related documents of the same type in recent years. This design has a wide working frequency band and can meet the requirements of a wide bandwidth. Considering the antenna volume, size, bandwidth, and peak gain, the target antenna is basically the best.

**Table 4:** Comparison of the proposed antenna to the same type of the antenna literatures.

References	Ant. size (mm^3^)	Antenna type	Freq. (GHz)	10 dB Bandwidth (%)	Peak Realized Gain (dBi)	Phantom Size in Measurement (mm^3^)	Link Budget of the Antenna?
^3^	20.5 × 30 × 0.05	Conformal	0.402	3.73	− 32.0	50 × 50 × 60	No
^4^	17.28 × 20 × 0.578	Conformal	2.45	12.2	− 35	–	No
^5^	π × (4.7)^2^ × 1.27	Circular	0.915	12.2	− 32.8	130 × 130 × 45.27	No
^6^	24 × 22 × 0.07	Flat	2.41	24.9	− 19.7	Height = 8	No
^7^	4 × 12 × 0.274	Flat	0.915	27.5	− 30.32	100 × 100 × 100	No
^8^	7 × 6 × 0.5	Flat	2.45	7.3	− 20.47	200 × 200 × 200	No
^9^	3 × 4 × 0.5	Flat	2.4	21.8	− 25.9	80 × 60 × 120	Yes
^18^	7 × 7 × 0.2	Flat	2.45	–	− 22	25 × 25 × 25	Yes
This work	3 × 3 × 0.5	Flat	2.45	22	− 24.9	60 × 60 × 150	Yes

## Conclusion

This paper introduces a compact and micro-implanted antenna used in wireless cardiac pacemaker systems. The miniaturized design of the antenna is realized by selecting a dielectric substrate with a high dielectric constant and a folded serpentine structure, and the size is controlled within a very small space. The use of the ground slot method further improves the antenna's radiation characteristics and optimizes the antenna's impedance matching. The antenna is ultra-small in structure with a volume of 4.5 mm^3^. The SAR of this ultra-small antenna meets the requirements defined by IEEE C95.1-1999. Miniaturization has always been the eternal pursuit of implantable antennas. This design adheres to the principle of ultra-small and compact structure and the original aspiration of meeting the antenna's radiation performance, providing a better reference solution for the antenna design of wireless cardiac pacemaker systems.
